# Effects of Oral Stimulation Intervention in Newborn Babies with Cri du Chat Syndrome: Single-Subject Research Design

**DOI:** 10.1155/2018/6573508

**Published:** 2018-05-08

**Authors:** Mi Kyung Kim, Deok Ju Kim

**Affiliations:** ^1^Department of Occupational Therapy, Sanggye Paik Hospital, Seoul, Republic of Korea; ^2^Department of Occupational Therapy, Health Science College, Cheongju University, Cheongju, Republic of Korea

## Abstract

The purpose of this study is to treat dysphagia in a newborn baby with cri du chat syndrome using an oral stimulation intervention and to examine its effects. The subject of this study was a baby born 2 weeks prematurely. Since birth, his oxygen saturation (SaO_2_) decreased while feeding, and he had difficulty with mouth feeding. Thus, an NG feeding tube was inserted, and dysphagia treatment was initiated on the sixth day after birth. A baseline phase and an intervention phase were performed using an AB design. The oral stimulation intervention was not performed in the baseline phase, as only nonnutritive sucking training using a rubber pacifier was used during the baseline phase. During the intervention phase, nonnutritive sucking training and oral stimulation intervention were simultaneously conducted. After the intervention period, daily oral milk intake and intake per feeding of the subject noticeably increased. The oxygen saturation while feeding rose over 90% on average, and the baby did not present with hypoxia. The oral stimulation intervention provided prior to feeding resulted in highly positive effects, including induced normal development of the baby, stimulation of his transition from the NG feeding tube to bottle feeding, increased oxygen saturation, and a shortened hospital stay.

## 1. Introduction

Cri du chat syndrome is a congenital genetic disorder resulting from the deletion of different sizes of the short arm of chromosome 5 (5p). It was first reported in 1963 by French doctor Jerome Lejeune who is renowned for his discovery of Down syndrome. One of the remarkable clinical symptoms of cri du chat is a characteristic cat-like cry. Affected children cry with a weak one-octave-higher minor key. Some cases reported an anomaly in a deformed larynx or tracheobronchomalacia. The crying sound barely changes through growth, but the frequency of the cry may decrease [1].

Clinical features vary considerably from patient to patient but childhood and distinct facial dysmorphism. Malocclusion, hyper- and hypotonia, and delayed motor development are also common as well as microcephaly. Affected children also present with hypertelorism, micrognathia, epicanthal folds, deformed low-set ears, palate problems x(high arch or very wide and flat in shape), hypotonia, abnormal palm lines, and intellectual disability. Birth weight is markedly low, the face is round, and the eyes are wide-set in these subjects. The prevalence is 1 in 15,000 to 50,000 births, and the rate is known to be higher in females than in males [2]. The age of the mother is not associated with the presence of the syndrome, but several studies reported that a history of radiation in prepregnancy, infection of influenza or mumps in early pregnancy, and medication used before or during the pregnancy may be the cause [3]. Many patients die during their early childhood. Most patients surviving to adulthood demonstrate an IQ level lower than 20. Typical characteristics include eating problems due to difficulty in swallowing and sucking from early childhood. Most children with the syndrome are born with a low weight at birth, showing poor growth. Their cognitive and linguistic abilities and development of motor nerves are significantly delayed. They demonstrate behavioural problems, including attention deficit hyperactivity disorder (ADHD), aggressiveness, and repetitive behaviour [4].

In the cri du chat syndrome, dysphagia is frequently observed in young children occurring since their neonatal period. The term dysphagia comprehensively includes all problems that can occur during the process in which food travels from the mouth to the stomach [5]. Patients with dysphagia mainly show functional disabilities in the oral and pharyngeal phases, such as residues in the pharynx and aspiration. This causes various complications including malnutrition, aspiration pneumonia, and dehydration, which can even cause death in some severe cases [6]. Dysphagia not only interrupts growth of the child but is also an important issue that might threaten one's survival.

Amongst premature and newborn babies, some show problems in oral feeding due to various causes, including a weakened cardiorespiratory system and oral muscles, problems in anatomical jaw structure, and overreaction to touching the mouth. In this case, a nasogastric feeding tube (NG feeding tube) is usually applied to initiate feeding. An NG feeding tube is a device used to improve the nutritional status of undernourished patients and to provide effective nutritional support. However, babies may be exposed to risks of side effects such as complications like aspiration, diarrhoea, and abnormal oral reflexes, as the period of using the NG feeding tube increases [7]. The transition to oral feeding is not easy for premature and newborn babies with weak swallowing abilities; thus, great time and sophisticated nursing care are required until they can handle oral feeding. Studies to investigate nonnutritive sucking and oral stimulation intervention, aiming at shortening the period of hospital stay by improving the oral feeding ability, should be conducted. In particular, oral stimulation intervention is an effective solution to improve dysphagia in premature and newborn babies [8, 9]. Oral stimulation therapy has been proposed as an effective measure in improving dysphagia of premature infants and newborns, but few studies reflect actual cases of interventions for premature infants and newborns that employ this method. This study is intended to help clinical therapists by providing guidelines for oral stimulation therapy and the effects of treatment.

There are only a handful of cases in existing studies that have investigated clinical cri du chat syndrome cases, due to its characteristic as a rare disorder. A few studies treated ophthalmological opinions and deformities to the genitourinary system. However, unfortunately, no study was found on neonatal dysphagia treatment. We reasoned that an occupational therapist is the most fitting therapist than any other specialists in treating dysphagia, and their role and intervention methods are absolutely critical. According to this law, “Dysphagia rehabilitation is calculated as training by one occupational therapist of a dysphagia patient in one-on-one sessions lasting at least 30 minutes” [10]. Therefore, this study presents the treatment and results of dysphagia in cri du chat syndrome patients by an occupational therapist using oral stimulation intervention.

## 2. Methods

### 2.1. Participants

#### 2.1.1. Medical History

**Figure 1:**
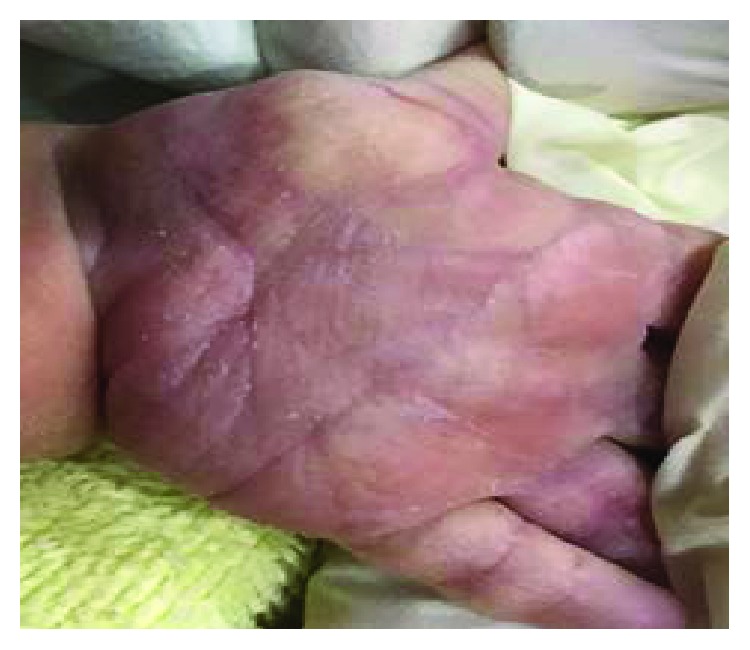
Single palm lines on both hands.

**Figure 2:**
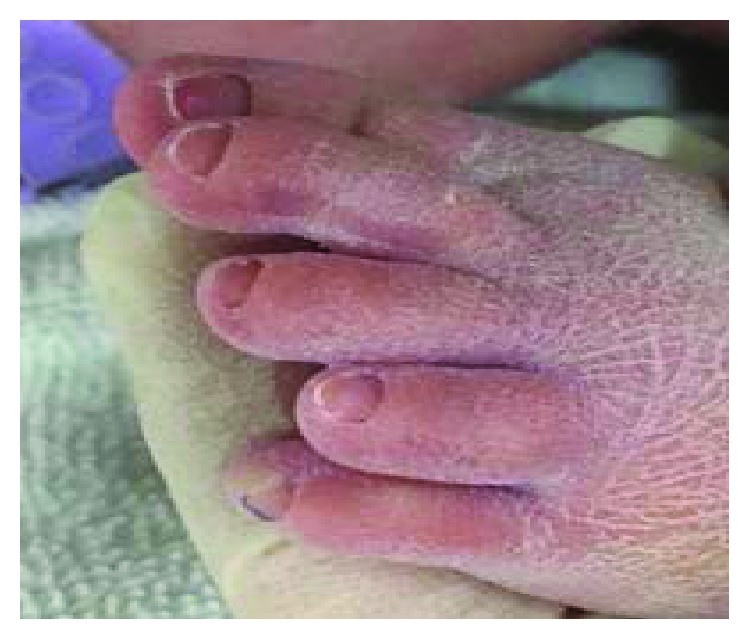
Protruding short fourth toe.

**Figure 3:**
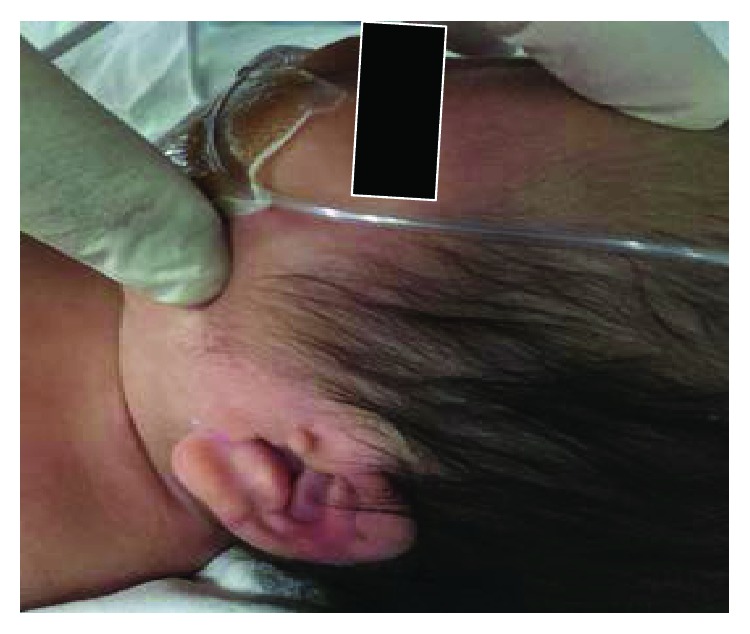
Both ears with a skin tag.

**Figure 4:**
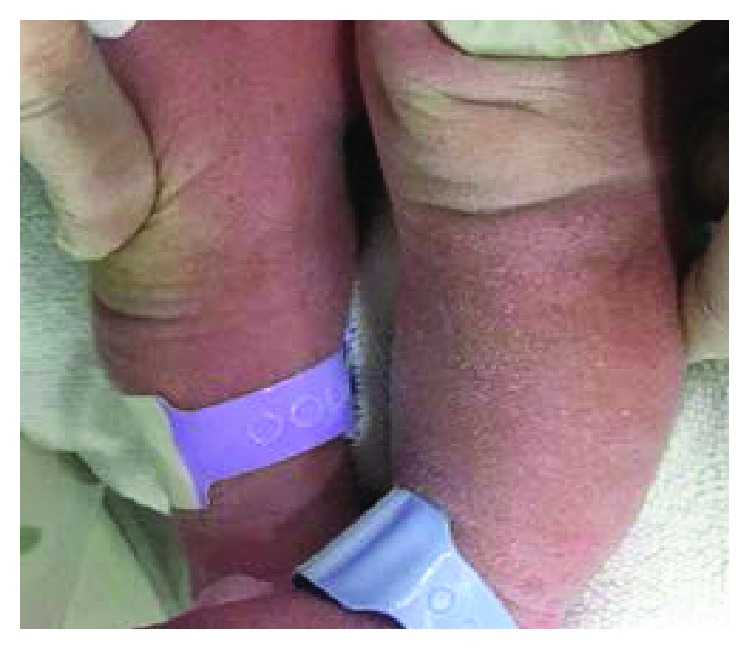
Left leg with a club foot.

**Figure 5:**
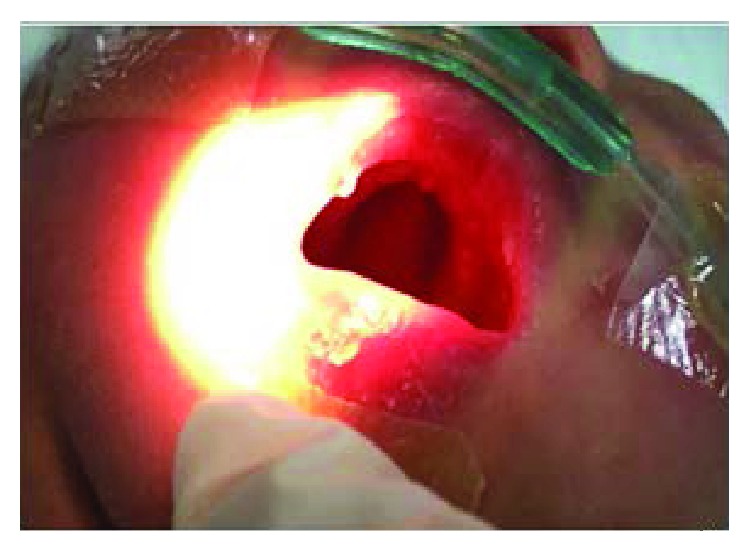
High-arched palate.

The baby patient included in this study was born at 37 weeks + 5 days weighing 3120 g via C-section according to the parents' requirements at P obstetrics on March 14, 2017. The oxygen saturation (SaO_2_) of the baby reduced at every feeding from the morning of his birth. His physical characteristics included a similar single palm line on both palms (Figure 1), a protruding short fourth toe (Figure 2), both hands rotated inwards, both ears showed a skin tag (Figure 3), a left club foot (Figure 4), and a high-arched palate (Figure 5). The baby was moved to S Hospital based in Seoul for more in-depth examinations. After the transfer, he was diagnosed with cri du chat syndrome based on his cat-like cry and physical defects. Cyanosis occurred while feeding due to shortness of breath, so an NG feeding tube was inserted. Swallowing therapy was requested to the department of rehabilitation medicine on March 20, 2017.

#### 2.1.2. Swallowing Function

Dysphagia was first evaluated 6 days after birth. In the initial evaluation, the baby received an NG feeding tube while on ventilator support. In oral motor function, the oral sensory ability was normal. However, his ability to close his lips and cheek and jaw stability were low due to weak facial muscle tone. Rooting reflex and bite reflex were intact, but his suck-swallow reflex was impaired. Sucking power was weak. Sucking, swallowing, and breathing were not smoothly coordinated. This reduced the oxygen saturation while feeding down to 72%. When the aspirator oxygen concentration was maintained at 21% (oxygen: 2 L/min, oxygen aspiration path: high flow), the oxygen saturation was recovered back to 90%. The possible amount per feeding was 5 cc per time.

### 2.2. Study Environment and Design

#### 2.2.1. Study Environment

The treatment of dysphagia was performed while the baby was inside the incubator in the Neonatal Intensive Care Unit (NICU) of the S Hospital. The environment was equipped with baby bottles for feeding, instruments for the NG feeding tube, in case of difficulty in mouth feeding, and a device to measure oxygen saturation. The subject of this study is a premature baby in the NICU. Hospitals do not use FEES and VFES because they can be dangerous to premature infants in the NICU. Although more objective evaluation results could be obtained using FEES or VFES, we could not use either given the condition of our subject.

#### 2.2.2. Study Design

This study is based on a single-subject research design and applied AB design. The study period was from March 15 to April 7, 2017. Twenty sessions were performed in total. During the baseline phase, nonnutritive sucking training (with a rubber pacifier) was initiated. A nurse provided nutrition using oral feeding or the NG feeding tube. During the intervention phase, 30 minutes of nonnutritive sucking training and another 30 minutes of oral stimulation intervention were performed, followed by oral or use of the NG feeding tube, which were equally performed at the baseline phase.

### 2.3. Instruments

#### 2.3.1. Evaluation of Feeding Amount: Baby Bottle

A Green mom PP bottle of 140 mL for newborn babies was used to measure the feeding amount in this study. The minimum unit of the bottle was 10 mL.

#### 2.3.2. Evaluation of Oxygen Saturation: Masimo Radical 7

**Figure 6:**
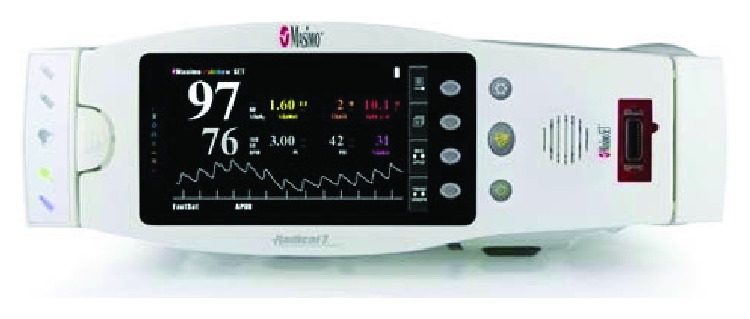
Masimo Radical 7.

To measure the oxygen saturation of the subject, a Signal Extraction Pulse CO-Oximeter manufactured by Masimo Radical 7 was used (Figure 6). Many studies found that this equipment significantly reduced false alarms and accurately detected actual warning signs. Its reliability was proven in more than 100 independent and objective studies. It is estimated to have been used for more than 100 million patients in major hospitals and medical institutions around the world. For measurement, the equipment records the evolution of oxygen saturation of the subject while feeding. An oxygen saturation falling below 80% indicates reduced ability to suck, swallow, and breathe, which is considered as hypoxia [11].

### 2.4. Study Procedure

#### 2.4.1. Baseline Phase

During the baseline phase, the baby was fed 40 cc every 3 hours. Oral feeding was first attempted with a baby bottle, and the remaining feeding sessions were provided by the use of the NG feeding tube. During this period, only nonnutritive sucking training was performed without oral stimulation intervention. The amount of oral milk intake fed with a bottle and the evolution of oxygen saturation while feeding were measured once a day, 5 times per week. To minimise error in measurement, no treatment stimuli were provided during this measurement. The same nurse fed the baby with milk inside the incubator at a specific hour to minimise any environmental factors that may have affected the baby's swallowing.

#### 2.4.2. Intervention Phase

During the intervention phase, nonnutritive sucking training and oral stimulation intervention were performed for 30 minutes each, and feeding was provided equally as during the baseline phase. The oral stimulation intervention was performed 5 times a week for 3 weeks (15 times in total). During this period, the feeding amount and oxygen saturation were measured daily after the oral stimulation intervention.

#### 2.4.3. Details of Intervention

The objective of dysphagia treatment is to help preterm infants' transition from feeding tube use to oral feeding. The stimulation program was designed in reference to the “oral stimulation program” conducted by Fucile et al. [12] and Choi et al. [13]. The program is performed for 30 minutes in each session and consists of stimulating the cheeks, upper and lower lips, upper and lower gums, internal cheeks, and the edge of the tongue, in that order (Table 1). Treatment of this program was performed by an occupational therapist with more than 10 years of experience in child dysphagia treatment and education. The activity level was adjusted during the program according to the condition of the baby.

### 2.5. Analysis Methods

Collected data were analysed with Microsoft Office Excel 2007. The feeding amount and oxygen saturation were checked daily during both the baseline and intervention phases. A frequency analysis was performed on the results. Measured values obtained during these phases were comparatively analysed with visual graphs and descriptive statistics to observe the evolution.

## 3. Results

### 3.1. Evolution of the Daily Oral Feeding Amount

**Figure 7:**
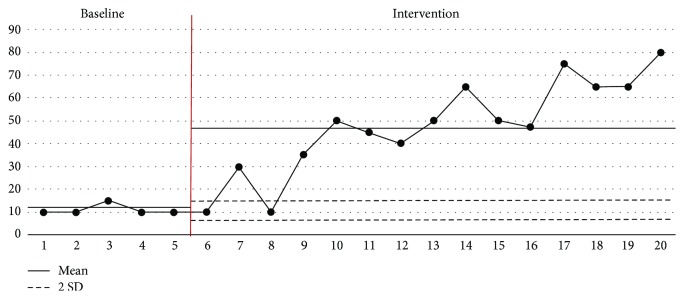
Evolution of the total amount of oral feeding.

The visual evolution of the daily oral feeding amount is presented in Figure 7. During the baseline phase, the mean and standard deviation values (mean ± SD) were 11.6 ± 2.0 mL. Nonnutritive sucking training and a 30-minute oral stimulation therapy were performed during the 15th session of the intervention phase. The mean ± SD values of the intervention phase were found to be 43.8 ± 22.5 mL, whereas the average value of the intervention phase increased by about 32.8 mL.

During the intervention phase, 13 of the 15 sessions showed results greater than +2 standard deviations of the baseline on average, except for the sixth and eighth sessions. This indicates that oral stimulation intervention had a positive effect on changes in the oral feeding amount.

### 3.2. Evolution of the Amount per Oral Feeding

**Figure 8:**
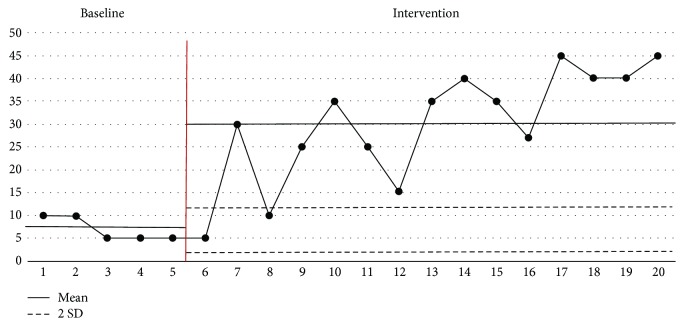
Evolution of the amount per oral feeding.

The progress of each oral feeding is presented in the graph in Figure 8. The mean ± SD of the baseline phase was 7.0 ± 2.5 mL, and the mean ± SD in the intervention phase was 27.1 ± 13.1 mL. That of the latter increased by 20.1 mL. During the intervention phase, 13 of the 15 sessions showed results greater than +2 standard deviations from the baseline on average, except for the sixth and eighth sessions. This shows that oral stimulation intervention had a positive effect on changes in the oral feeding amount.

### 3.3. Evolution of Oxygen Saturation

**Figure 9:**
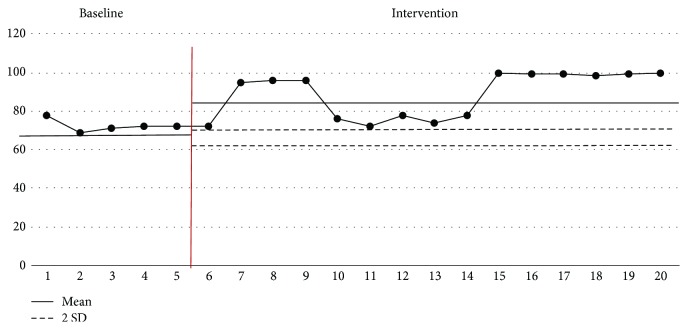
Evolution of oxygen saturation.

The evolution of oxygen saturation while feeding is presented in Figure 9. The mean ± SD of the baseline phase was 72.4 ± 3.0%, and that of the intervention phase was 90.7 ± 10.9%, whereas the latter increased by 18.3%. During the intervention phase, all 15 data values were greater than +2 standard deviations of the baseline on average, which indicates that the oral stimulation intervention had a positive effect on the increase of oxygen saturation while feeding. The resulting values demonstrate that those of oxygen saturation from the tenth to fourteenth sessions fell lower than the average value. This is because the baby went through difficulties in adaptation as the aspirator was completely removed, and he breathed on his own while fed after the tenth session. Afterwards, the baby gradually adapted to self-breathing. The oxygen saturation increased while feeding because of the oral stimulation intervention program.

## 4. Discussion

Cri du chat syndrome is a disorder caused by the loss of the chromosome 5p's short arm, which causes characteristics physical birth defects and the typical cat-like cry. In the neonatal period, neonates with cri du chat syndrome present difficulty in feeding and shortness of breath. In the childhood stage, they may present with frequent upper airway infection, otitis media, and diarrhoea, often requiring internal medicine treatment [14]. The feeding problem, which is particularly frequent during the neonatal period, is a serious problem that can even threaten the survival of the baby; therefore, dysphagia treatment during this phase is highly critical.

The subject of this study showed dysphagia and decreased oxygen saturation while feeding since the early neonatal period, and dysphagia treatment was initiated on the sixth day after birth. In particular, reduced oxygen saturation while feeding can cause instability in the cardiovascular system, respiratory system, and central nervous system, formation of an immature oral structure, and other problems [15]. An uncoordinated suck-swallow-breath pattern hinders safe and successful oral feeding. If proper breathing is not maintained in between sucking, owing to the dysfunctional coordination of sucking, swallowing, and breathing, the infant may suffer reduced oxygen saturation. Repeated hypoxia can damage the cerebrum by reducing blood flow to the cerebrum. Delayed development of the oral feeding ability can lead to growth retardation [14].

Various studies on oral stimulation intervention for improving oral feeding ability have been conducted [8, 9]. This study also employed an oral stimulation program to verify whether such programs are truly effective for dysphagia. This method stimulated the cheeks, upper and lower lips, upper and lower gums, internal cheeks, and the edge of the tongue to treat dysphagia in premature and neonatal babies. The duration and frequency of stimulation was properly adjusted for each phase. This study conducted an oral stimulation intervention program to a baby with cri du chat syndrome for 4 weeks according to our manual. The study was a single-subject research design and applied AB design. Only nonnutritive sucking training was performed during the baseline phase. During the intervention phase, nonnutritive sucking training and a 30-minute oral stimulation intervention were simultaneously performed.

The average daily intake of the subject was 11.0 ± 2.0 mL in the baseline phase and 43.8 ± 22.5 mL in the intervention phase, in which the latter increased by 32.8 mL. As for evolution of intake per feeding, the average of the baseline phase was 7.0 ± 2.5 mL, and that of the intervention phase was 27.1 ± 13.1 mL, which increased by 20.1 mL. Fucile et al. [16] conducted an experiment on 32 premature babies to investigate the effect of oral stimulation intervention. The experimental group moved on to bottle feeding from an NG feeding tube 7 days earlier with a greater feeding amount compared to the control group, showing a positive effect. Boiron et al. [17] found that the feeding amount increased when oral stimulation intervention was performed immediately before feeding. The increased amount of feeding led to an increased body weight, and the in-hospital period shortened. Their results support those of this study. In respect to the evolution of oxygen saturation, the average of the baseline phase was 72.4 ± 3.0%, and the intervention phase showed 90.7 ± 10.9%, with the latter increasing by 18.3%. Feeding is a complex process that requires exact harmonization of sucking, swallowing, and breathing, and it is difficult to increase the feeding amount steadily if breathing is not good. This has been proven in several studies. The increase in the subjects' feeding amount in this study indicates that the functions of sucking, swallowing, and breathing have improved in the end, which is attributed to the effects of oral stimulation. [18]. Choi et al. [13] reported that sucking frequency increased when the mouth area was stimulated during oral feeding. They also observed that this increased oxygen saturation stabilized the patient's behavioural status.

Finger sucking strength of the baby gradually increased compared to the beginning, as the intervention frequency increased. Amaizu et al. [19] observed that the complete bottle feeding was attained earlier when the first bottle feeding was started earlier. The bottle feeding amount increased as the baby's sucking ability developed. This probably functioned as positive feedback to the baby and improved the sucking ability even further. Intervention occurring on a continued and regular manner particularly stimulates more positive results [12]. This study regularly provided stimulation at a specific hour every day, which enabled us to observe the baby more closely.

The limitations of this study are as follows. First, this study was limited as a single-subject design due to the characteristics of the cri du chat syndrome, which is a rare disease. This makes it difficult to generalise the effects of intervention to all babies with cri du chat syndrome. Second, this study does not include a follow-up period that allows continued examination of the treatment effect after the end of the intervention phase. It is recommended that future studies shall investigate the continuity of treatment effects using an ABA research design. This study is meaningful because it conducted an oral stimulation intervention program on a baby affected by the cri du chat syndrome. Currently, there are few domestic and international cases of feeding intervention to premature and neonate babies to assist with their normal development. The study offers meaningful implications as it stimulated the transition from the NG feeding tube to bottle feeding and confirmed that the program was a highly effective treatment intervention that augmented oxygen saturation.

## 5. Conclusion

This study presented the process and results of dysphagia treatment to a baby with cri du chat syndrome through an oral stimulation intervention program led by an occupational therapist. We employed an AB design and determined a baseline phase and an intervention phase. The oral stimulation intervention was not provided during the baseline phase, and nonnutritive sucking training using a rubber pacifier was performed. During the intervention phase, the nonnutritive sucking training and the oral stimulation intervention were conducted simultaneously.

In terms of the intervention results, the daily intake and the intake per feeding noticeably increased in the intervention phase compared to the baseline phase. The oxygen saturation from the morning of the day of birth to the baseline phase decreased at every feeding (the rate was lower than 80% on average, and the baby presented with hypoxia). After the intervention phase, this value rose to over 90% on average. The oral stimulation intervention conducted prior to feeding promoted normal development of the baby, demonstrating highly positive effects, including stimulation of the transition from the NG feeding tube to bottle feeding, increased oxygen saturation, and reduced hospital stay.

## Figures and Tables

**Table 1:** Oral stimulation program.

Structure	Stimulation steps	Frequency	Duration
Cheek	(1) Place the index finger at the base of the nose. (2) Compress the tissue, move finger toward the ear, then down, and toward the corner of the lip (in a C pattern). (3) Repeat for the other side. (i) Purpose: improve the range of motion and strength of cheeks and improve lip seal	4x each cheek	2 min

Upper lip	(1) Place the index finger at the corner of the upper lip. (2) Compress the tissue. (3) Move the finger away in a circular motion, from the corner toward the centre, and to the other corner. (4) Reverse direction. (i) Purpose: improve the lip range of motion and seal	4x	1 min

Lower lip	(1) Place the index finger at the corner of the lower lip. (2) Compress the tissue. (3) Move the finger away in a circular motion, from the corner toward the centre, and to the other corner. (4) Reverse direction. (i) Purpose: improve the lip range of motion and seal	4x	1 min

Upper and lower lip curls	(1) Place the index finger at the centre of the upper lip. (2) Apply sustained pressure and stretch downward toward the midline. (3) Repeat for the lower lip, apply sustained pressure, and stretch upward toward the midline. (i) Purpose: improve the lip strength, range of motion, and seal	2x each cheek	1 min

Upper gum	(1) Place a finger at the centre of the gum, with firm sustained pressure slowly move toward the back of the mouth. (2) Return to the centre of the mouth. (3) Repeat for the opposite side. (i) Purpose: improve the range of motion of the tongue, stimulate swallowing, and improve sucking	2x	1 min

Lower gum	(1) Place a finger at the centre of the gum, with firm sustained pressure slowly move toward the back of the mouth. (2) Return to the centre of the mouth. (3) Repeat for the opposite side. (i) Purpose: improve the range of motion of the tongue, stimulate swallowing, and improve sucking	2x	1 min

Internal cheek	(1) Place a finger at the inner corner of lips. (2) Compress the tissue, move back toward the molars, and return to the corner of the lip. (3) Repeat for the other side. (i) Purpose: improve the cheek range of motion and lip seal	2x each cheek	2 min

Lateral borders of the tongue	(1) Place a finger at the level of the molar between the side blade of the tongue and the lower gum. (2) Move the finger toward midline, pushing the tongue toward the opposite direction. (3) Immediately move the finger all the way into the cheek, stretching it. (i) Purpose: improve the tongue range of motion and strength	2x each cheek	1 min

Midblade of the tongue	(1) Place an index finger at the centre of the mouth. (2) Give sustained pressure into the hard palate for 3 seconds. (3) Move the finger down to contact the centre blade of the tongue. (4) Displace the tongue downward with a firm pressure. (5) Immediately move the finger to contact the centre of the mouth at the hard palate. (i) Purpose: improve the tongue range of motion and strength, stimulate swallowing, and improve sucking	4x	1 min

Elicit a suck	(1) Place a finger at the midline, centre of the palate, gently stroke the palate to elicit a suck. (i) Purpose: improve sucking and soft palate activation	N/A	1 min

Pacifier	(1) Place a pacifier in the mouth. (i) Purpose: improve sucking and soft palate activation	N/A	3 min
